# Protective eye device for eyelid brachytherapy: Technical report of an innovative personalized method

**DOI:** 10.1097/MS9.0000000000003211

**Published:** 2025-04-15

**Authors:** Wijdane El Hawari, Sanaa El Majjaoui, Oussama Bentahar

**Affiliations:** a Faculty of Medicine, Pharmacy and Dental Medicine of Fez, Department of odontological sciences, Sidi Mohamed Ben Abdellah University, Fez, Morocco; b Department of Radiotherapy, University Mohammed VI of health sciences, Casablanca, Morocco; c Faculty of Medicine, Pharmacy and Dental Medicine of Fez, Department of odontological sciences,Sidi Mohamed Ben Abdellah University, Fez,Morocco

**Keywords:** basal cell carcinoma, eyelid brachytherapy, eye protection device, radiotherapy

## Abstract

**Introduction and importance:**

Carcinoma of the eyelid is a specific anatomical variant of skin cancer, notable for its proximity to the eye. Surgery is the gold standard for managing eyelid cancer, while radiotherapy can serve as an alternative for patients unfit for surgery or as an adjunct treatment. Various devices in maxillofacial prosthodontics are used in conjunction with head and neck radiotherapy to protect the healthy tissues surrounding the irradiation site. These devices are designed by a maxillofacial prosthodontics specialist in collaboration with a radiotherapist/oncologist.

**Case presentation:**

This technical report presents an innovative and original method for an eye protection device against radiation. We describe a case involving a 60-year-old woman with basal cell carcinoma of the eyelid, initially treated with surgical excision. High-dose rate brachytherapy was indicated, with a total dose of 40 Gy administered in 8 fractions. Prior to treatment delivery, it was necessary to protect the eyeball from the adverse effects of eyelid BT. We devised a novel device to shield the eyeball and its adnexa.

**Clinical discussion:**

This article details the technique for the creation and insertion of this device. To our knowledge, this is the first reported case of an intraocular protective device made with lead. This invention was patented under the reference [MA 56825 B1].

**Conclusion:**

The use of a stent designed to shield adjacent tissues from the side effects of radiotherapy is highly advantageous, particularly for critical sites such as the one presented in this clinical case. The success of eye protection in this instance is encouraging and suggests the potential to expand the application of similar devices.

## Introduction

Skin cancer is one of the most widespread cancers, including basal cell carcinoma, squamous cell carcinoma, melanoma, and nonepithelial skin cancer^[^1^]^.

Carcinoma of the eyelid is a specific anatomical variant of skin carcinoma, characterized by rare lymph node invasion and very low rates of metastasis. The proximity of the eyeball and its adnexa complicates treatment^[^2^]^.

The therapeutic options for its management include conventional surgical intervention, Mohs micrographic surgery, cryosurgery, external beam radiation therapy, high-dose-rate brachytherapy (BT), and electronic BT^[^1^]^. Surgery remains the gold standard. Radiotherapy can be an alternative for patients unfit for surgical intervention^[^3^]^ or additional treatment in case of poor histological prognostic factors.

**Highlights**

- **Purpose and scope**: Carcinoma of the eyelid, a specific skin cancer variant near the eye, is primarily managed through surgery. Radiotherapy is an alternative for those unable to undergo surgery or as an adjunct therapy. Specialized maxillofacial prosthodontic devices help protect healthy tissues during radiotherapy.
- **Innovative device**: The technical report introduces a new eye protection device designed for radiation therapy. This device is custom-molded from the patient’s eye impression and contains lead to protect the eye during brachytherapy and external radiotherapy.
- **Collaboration**: The development of this device involves collaboration between maxillofacial prosthodontics specialists and radiotherapists/oncologists, ensuring the device meets the specific needs of radiation treatment for eye protection.
- **Advancement in treatment**: The proposed device represents a significant innovation over existing options, aiming to improve the protection of the eye and adjacent tissues during radiotherapy for cervicofacial cancers.
- **Encouraging results**: The device’s successful application in the presented clinical case demonstrates its effectiveness in shielding critical sites from radiotherapy side effects, suggesting potential for broader use in similar treatments.

The ultimate goals in treating eyelid cancer are local tumor control without compromising aesthetic outcomes^[^2^]^.

BT delivers a high concentration of radiation dose to the tumor with a rapid dose drop over a few millimeters from the radioactive source^[^3^]^. It can be a better therapeutic option; interstitial BT with 192Ir for carcinoma of the eyelid and inner canthus allows precise implantation in these small areas, providing a high local cure rate while maintaining function and achieving good aesthetic results, with acceptable late toxicity^[^4^]^.

High-dose rate (HDR) BT is described for treating basal cell carcinomas and squamous cell carcinomas of the lower eyelid via superficial catheters^[^5^]^.

Some studies have reported complications of BT, such as epiphora, pigmentation changes,^[^6-8^]^ lacrimal duct stenosis^[^9^]^, impairment of the eyelid fissure^[^8^]^, ectropion, cataract, conjunctivitis, keratoconjunctivitis sicca, eyelid malocclusion, hyperlacrimat ion, and small corneal ulcer^[^7,9^]^.

In maxillofacial prosthodontic practices, various types of devices are used in conjunction with head and neck radiotherapy to protect the healthy tissues surrounding the irradiation site.

These devices are designed by a maxillofacial prosthodontics specialist in collaboration with a radiotherapist/oncologist^[^10^]^.

Following this line of thought, we devised a device to protect the eyeball and its adnexa from the adverse effects of eyelid BT. This device is intended to cover the eye for protection.

We present, through this clinical case, an original technique for fabricating a protective eye device by describing the prosthodontic and laboratory steps and the protocol for its positioning before and during BT treatment. This invention was patented under the reference [MA 56825 B1].

This work has been conducted and reported in accordance with the SCARE 2023 guidelines, ensuring adherence to the established criteria for surgical case reports. The methodology and structure follow the SCARE 2023 standards, as outlined in the referenced paper^[^11^]^.

## Materials and methods

### Clinical history

A 60-year-old woman was referred to the National Institute of Oncology in Rabat, Morocco. The history of the disease dates back to March 2021, when a small nodule appeared at the outer canthus of her left eye. The patient first consulted a dermatologist, who then referred her to the maxillofacial surgery department, where she underwent a biopsy excision. The biopsy revealed a basal cell carcinoma measuring 0.8 cm in its largest dimension, with tumoral involvement at the internal margin.

**Figure 1.**
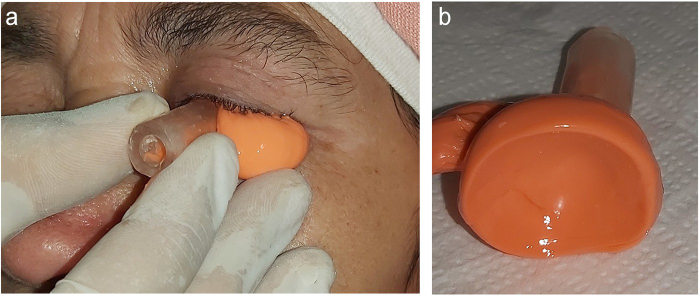
Impression technique: (a) fixation of the mixing silicone tube with the special try impression, (b) acquired ocular surface.

Subsequently, the patient was referred to the oncological radiation department for adjuvant treatment. During consultation, the patient was classified as OMS grade 0, exhibited a good skin healing process, and showed no signs of inflammation (Fig. 1).

The proposed treatment was HDR BT using the Freiburg flap applicator, with a total irradiation dose of 40 Gy, delivered at 5 Gy per session over 8 sessions, with 2 or 3 sessions per week.

### Fabrication of the intraocular leaded protective stents

**Figure 2.**
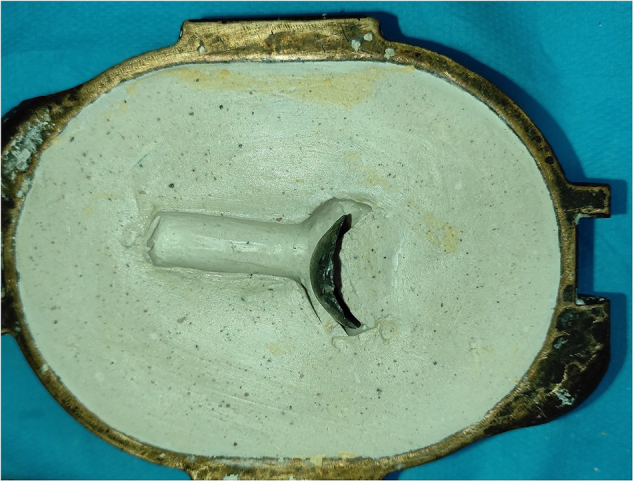
Elaboration technique: Lead positioned on the flask.

**Figure 3.**
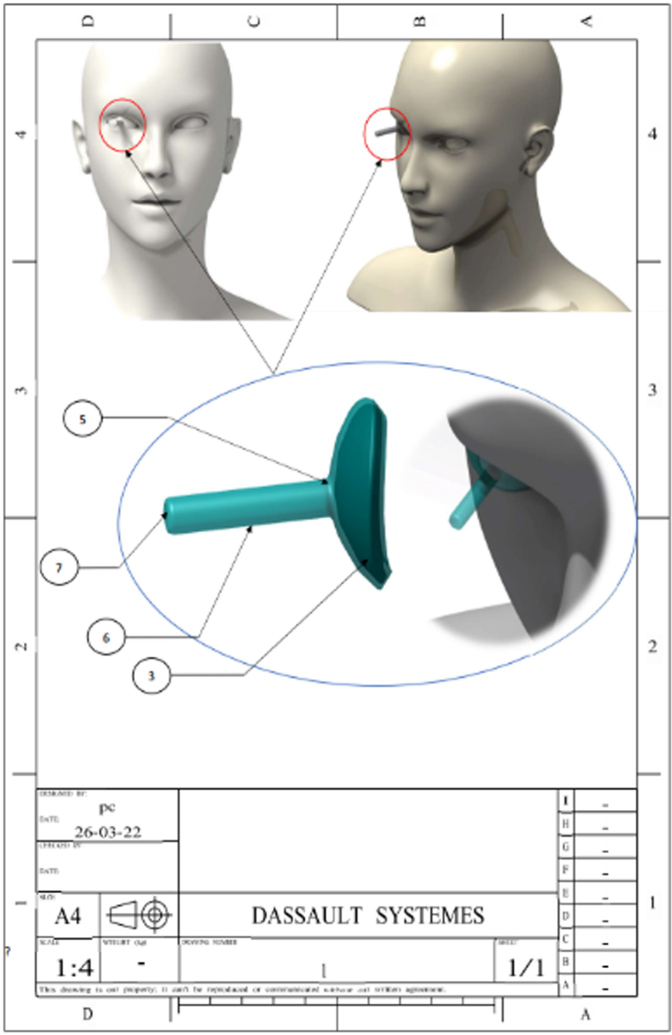
Schematic representation of the device.

The procedure was carried out in the Department of Prosthodontics at the Center of Consultations and Dental Treatments of Rabat, Morocco. A special tray impression (Fig. 2) was created using a resin hull and a mixing silicone tube, following the realization of the silicone mold. The tray was then tempered and inserted into the eye. Polyvinyl siloxane light body was injected into the tissue surface for accurate surface detail reproduction. After approximately 5 minutes, the impression was removed. It was then placed in the lower half (Fig. 3a) of the flask, which was filled with type II gypsum material. The flask was closed by placing the upper half part and compressing it, resulting in a mold of the impression.

A standard lead block was adjusted to a model molding the extrados of the eyeball (Fig. 3b). This leaded hemisphere was delicately inserted into the lower half of the flask. Its position was maintained by covering the lateral parts of either side of the plate with transparent resin (hard consistency).

**Figure 4.**
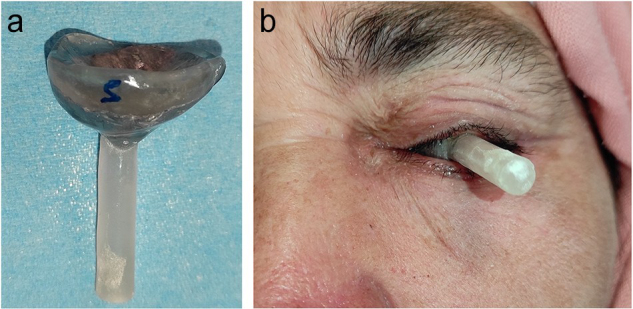
View of the leaded protective ocular stents: (a) stents after polishing and definition, (b) fitting step.

Clear polymethylmethacrylate was mixed and placed in the mold space. A curing cycle was carried out, maintaining a temperature of 70°C for approximately 2 hours. After this, the stent was removed from the flask. The gripping part was adjusted, finished, and polished. The device was then cleaned with a brush, liquid soap, and water, and disinfected (Fig. 4a). It was tried and adjusted for easy positioning and removal (Fig. 4b).

### Brachytherapy, insertion of the device, and follow up

**Figure 5.**
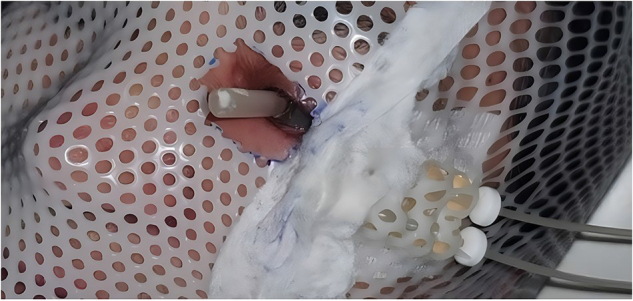
Brachytherapy simulation session: device insertion.

During a simulation BT session, the thermoformed mask was adjusted to fix the Freiburg flap applicator, and a clearance zone was created adjacent to the left eye (Fig. 5a, b). The treatment itself was carried out by repeating the steps performed during the simulation. The patient tolerated the treatment well, without developing acute skin toxicities. At the first follow-up consultation after 3 months, good local control was observed, with no late toxicities such as telangiectasias or skin fibrosis.

An ophthalmologist’s examination at 3 months reported no adverse reactions to the left eye protected by the device.

### Results and discussion

BT is a well-established approach for treating skin tumors.^[^12^]^. It is a minimally invasive radiation technique that provides a high level of local control and good cosmetic results for facial peri-orificial skin carcinomas^[^9,13^]^.

Some studies have reported acute and late ocular toxicities^[^3^]^ the idea of protecting the eyeball is very appealing and may significantly reduce the side effects of radiation treatment.

To our knowledge, this is the first reported case of ocular leaded protection. Other devices have been described in association with oral radiation therapy, designated to treat lesions of the buccal mucosa, skin, alveolar ridge, unilateral parotid, retromolar regions, palate, maxillary sinus, pharynx, tonsillar pillars, floor of the mouth, and tongue^[^14^]^.

Among these devices are perioral cone positioning stents, shielding oral stents, and tissue positioning stents, which are used to adjust the position and/or help protect uninvolved tissues from the side effects of radiation^[^15^]^.

On the other hand, some devices are used as vectors for the radioactive source, allowing the distribution of a concentrated dose of radiation to a specific area using capsules, tubes, seeds, or needles^[^14^]^. HDR BT with customized applicators for facial skin and scalp lesions has been noted to be efficient and safe^[^12^]^.

Concerning the protective stent described in this clinical case, the technique used is simple, inexpensive, and prevents severe complications. Dental impression materials, such as silicones, have been successfully used to register the contour of the eye socket^[^16^]^, and we easily adjusted the lead block to the impression model. Other low-temperature melting alloys, such as Wood’s metal (Cerrobend alloy), can also be used as an alternative to the lead block; it is commonly used to protect tissues not involved in the irradiation field^[^17^]^ and requires an additional step of casting after melting.

This device is extremely important. Due to the complexity of anatomical needs, specific radiation angles, and the HDR, the device helps reduce the cumulative dose the eye receives during HDR BT thanks to its personalized and specialized design. In comparison with the use of conventional protective glasses, which are effective in certain situations (such as conventional low-dose radiotherapy), they do not provide optimal protection for the eye against targeted doses in HDR radiotherapy, due to the HDR and the size of the glasses, which may interfere with the treatment area.

Moreover, based on the therapeutic index (the ratio between tumor control and consideration of at-risk organs), this device allows the radiotherapist to recommend HDR BT (thanks to this optimal protection) and thus optimize the chances of therapeutic success. Indeed, in the absence of an effective protection tool, the radiotherapist might be forced to opt for conventional radiotherapy as a compromise, which would reduce the chances of tumor control. Therefore, with this personalized and specialized protection, the chances of therapeutic success could be significantly improved. Furthermore, this device, thanks to its customized design, helps reduce or even prevent the immediate and late side effects of HDR BT, which are primarily:

Immediate complications: eye Irritation, corneal Damage, conjunctivitis (Pink Eye), swelling and edema.

- Late complications: dry Eye Syndrome, cataract Formation, retinal Damage, permanent Changes in Eyelid Function, late-onset conjunctival changes.

It is also worth noting that this device can be used for orthovoltage X-ray therapy and electron therapy, not only BT. Generalizing the use of such a device to other body locations seems promising. On the other hand, some devices are used as vectors for the radioactive source, allowing the distribution of a concentrated dose of radiation to a specific area using capsules, tubes, seeds, or needles. HDR BT with customized applicators for facial skin and scalp lesions has been noted to be efficient and safe.

The applicability of this ocular shield in other areas of radiotherapy may be wide, and we believe there should be further study beyond this initial study. This device could be adapted to other radiotherapies in the future, where protecting the eyes is crucial. For instance, in external beam radiotherapy for neoplasm located near the ocular structures, the shield could enhance protection from secondary radiation. In addition, while the ocular shield could be in the form of a device specifically designed for usage to the individual patient based on their anatomical characteristics through methods like 3D printing post impression taking or imaging-guided planning. There may also be advancements that merge the ocular shield with other protective technologies, like enhanced radiation filtration systems or real-time biofeedback.

Another interesting perspective is to use an integrated dosimetry sensor within the device to assess the dose received by the protected organ and the treated area. This would enable a quantitative evaluation of the case, monitoring, and comparative studies on the effectiveness of the device.

Though we believe strongly in the promise of this ocular guard, there are many limitations that must be acknowledged and overcome for future investigations. Even if such personalization at the level of specific impressions ensures an adequate fit and stabilization of the device for the single radiotherapy sessions, it can also create comfort-related problems, mainly during longer treatment periods. These devices must be precisely engineered so collaboration with a maxillofacial prosthodontist and the careful taking of impressions to produce accurate patient devices are required for design and fabrication as well. In our work we collaborated with prosthodontists, radiotherapists and physicists to make the ocular shield efficient from a radiological point of view while being perfectly adapted to the morphology of each patient.

Furthermore, although preliminary results are promising, additional clinical studies are necessary to validate the efficacy of the ocular shield in diverse patient populations and under varying treatment conditions. Larger multicenter trials would be needed to have long-term results and address potential biases. In conclusion, while our ocular shield holds considerable potential for improving eye protection during HDR BT, continued research is essential to overcome these limitations and fully explore the future applications of this device in the field of radiotherapy.

## Conclusion

Using a device designed to protect adjacent tissues from the side effects of radiotherapy is highly advantageous, particularly for critical sites such as the one presented in this clinical case. The success in eye protection is encouraging and suggests expanding the application of similar devices.

## Data Availability

Not applicable.
